# Comparative Visual Outcomes of Toric and Nontoric Intraocular Lenses Featuring Combined Diffractive Extended Depth of Focus and Multifocal Designs

**DOI:** 10.1155/joph/3788361

**Published:** 2026-07-02

**Authors:** Laureano A. Rementería-Capelo, Inés Contreras, Laura Mariñas-García, Vanesa Blázquez, Javier Ruiz-Alcocer

**Affiliations:** ^1^ Clínica Rementería, Madrid, Spain, clinicarementeria.es; ^2^ Instituto Ramón y Cajal de Investigaciones Sanitarias (IRYCIS), Hospital Universitario Ramón y Cajal, Madrid, Spain, hrc.es; ^3^ Optics and Optometry Department., Complutense University of Madrid, Madrid, Spain, ucm.es

## Abstract

**Purpose:**

To assess and compare visual quality and subjective outcomes of the toric and nontoric versions of a trifocal intraocular lens (IOL) combining a diffractive extended depth of focus (EDoF) profile with a diffractive multifocal pattern.

**Methods:**

This prospective cohort study included patients bilaterally implanted with the TECNIS Synergy or TECNIS Synergy Toric II IOL. At 3 months postoperatively, monocular and binocular uncorrected distance visual acuity (UDVA), corrected visual acuity (VA) at 100%, 50%, and 10% contrast, and binocular defocus curves were evaluated. A five‐point Likert scale questionnaire assessed satisfaction at different distances, perception of halos, and willingness to choose the same lens again.

**Results:**

A total of 114 eyes (52 nontoric and 62 toric) were analyzed. Monocular UDVA was 0.02 ± 0.07 logMAR (nontoric) and 0.08 ± 0.11 logMAR (toric) (*p* = 0.02), while binocular UDVA was −0.05 ± 0.06 logMAR and −0.02 ± 0.10 logMAR, respectively (*p* = 0.13). Defocus curves showed VA better than 0.1 logMAR from +1.0 to −2.5 D in both groups. No significant differences in contrast sensitivity were observed between groups at any level (*p* > 0.05). Over 80% of patients in each group reported being satisfied or very satisfied at all distances. Halos were described as not or only slightly bothersome by 42% of patients. A small percentage expressed a low likelihood of choosing the same lens again (4% nontoric, 9% toric).

**Conclusion:**

Both IOL models provided similar and effective visual restoration from distance to near. Despite some reported visual disturbances, patient satisfaction and visual quality were high and comparable between the toric and nontoric lenses when appropriately aligned.

## 1. Introduction

Multifocal intraocular lenses (IOLs) have succeeded in restoring vision at different distances, from the introduction of bifocal designs to the emergence of trifocal models. The latter have effectively improved visual acuity at intermediate distances, creating a more continuous range of vision from distance to near focus [1, 2]. Similarly, toric versions of these multifocal lenses, when properly implanted, have achieved comparable outcomes in patients with preoperative astigmatism [3–5].

While earlier designs employed refractive, diffractive or a combination of both optics to achieve multifocality, most current multifocal IOLs utilize diffractive designs. As previously mentioned, these designs restore visual acuity across a range of distances; however, some authors have reported that the optical properties and intraocular light distribution across multiple focal points may be associated with an increased incidence of dysphotopsia phenomena reported by patients [6]. To provide vision at different distances and reduce photic phenomena, novel designs of extended depth of focus (EDoF) IOLs have been developed. Rather than creating different foci into the eye as it happens with diffractive IOLs, EDoF lenses elongate a single focus avoiding overlapping multiple foci. It has been reported that while trifocal toric IOL still provide better vision at near distances, the effect of photic phenomena may be lower with EDoF IOLs [7].

Despite the satisfactory results, the industry continuously introduces new multifocal designs to improve patients’ visual quality. In this regard, the TECNIS Synergy (Johnson and Johnson Vision) and its toric version, TECNIS Synergy Toric II IOL, present a multifocal lens that does not feature a conventional diffractive design but is instead defined as a hybrid presbyopia‐correcting IOL. These designs combine multifocal and EDOF technologies within a proprietary diffractive surface [8, 9]. Some authors have reported that the range of vision provided by the nontoric version of the lens is satisfactory, similar to and potentially greater than other similar lenses currently available on the market [10–13]. Regarding dysphotopsias, some authors report that they are minimal [14], while others have consistently described the presence of adverse phenomena associated with their use [8, 9, 11].

Although there is currently available information regarding the nontoric version of this lens, there is limited evidence concerning the visual performance of the toric design. One recent study compared the toric version of the Synergy IOL with other trifocal toric IOL [15]. However, to the best of the author’s knowledge, no studies have reported the performance of the TECNIS Synergy IOL and compared the results to its toric version. Therefore, the aim of this study was to analyze and compare the visual function and satisfaction of patients that were bilaterally implanted with either the spherical or the toric version of this platform.

## 2. Patients and Methods

This prospective cohort study was performed in Clínica Rementería, Madrid, Spain, and considered healthy patients > 40 years that underwent routine cataract surgery with bilateral implantation with the nontoric or the toric version of the same IOL’s platform: TECNIS Synergy or the TECNIS Synergy Toric II IOL. Toric IOLs were implanted in patients with 0.8 D or more preoperative corneal astigmatism, and patients were considered for the study only if they were scheduled for binocular implantation of the same IOL’s version.

Exclusion criteria included amblyopia, previous ocular surgery, and presence of ocular pathologies and abnormal irises. At the same time, patients with intra‐ or postoperative complications will be excluded from the analysis of visual function.

To assess the criteria for the study, all subjects underwent an ophthalmologic examination, which included refraction, screening for ocular conditions and/or systemic diseases, slit‐lamp biomicroscopy, and fundus examination.

The study followed the tenets of the Declaration of Helsinki and was reviewed and approved by the pertinent ethics committee. Informed consent was obtained from all patients after the nature of the study had been explained.

### 2.1. Clinical Assessment

As in previous similar studies, the preoperative protocol included uncorrected distance visual acuity (UDVA), corrected distance visual acuity (CDVA), slit lamp evaluation of the anterior segment, intraocular pressure measurement, specular biomicroscopy (CEM‐530, NIDEK CO., LDT, Japan), dilated fundus examination, optical coherence tomography examination of the macula and optic nerve (Cirrus HD‐OCT 5000, Carl Zeiss Meditec AG, Germany), corneal measurements and IOL calculation with the IOL Master 700 (Carl Zeiss Meditec AG, Germany) [16]. The targeted refraction was *emmetropia* in all cases. Candidates to toric IOL implantation were also explored with the ARGOS biometer (Alcon Laboratories, USA). The power of the toric IOLs and their implantation axis were calculated with the ESCRS calculator, available on‐line at https://iolcalculator.escrs.org/. Spherical power was taken from the IOL master report and, following the manufacturer’s indications, the first IOL with the lowest positive spherical equivalent was chosen; the corneal values employed were those provided by the IOL master. The Barrett formula was employed, with centroid surgical‐induced astigmatism of 0.1 for right eyes and 0.15 for left eyes. For eyes with oblique astigmatism, the Holladay 1 formula was selected.

Cataract surgeries in the two groups were carried out by two experienced surgeons (L.A.R. and L.M.G) following a 2.2 mm clear‐cornea incision at 135°. The standard protocol for the surgery has been previously reported in other studies performed in our clinic [16]. In addition, toric IOL implantation was guided by the VERION System (Alcon Laboratories, USA).

### 2.2. Postoperative Assessment

The evaluation of the patients was performed one day after each procedure, 1 week, 1 month, and 3 months after the second eye surgery, but only results of the 3‐month visit were considered for the study. For the monocular analysis, the right eye of each patient was selected.

In the 3 month follow‐up visit, monocular and binocular UDVA was measured using a 22″ LED liquid crystal display system (CC‐100 HW 5.0 Series, Topcon) that can display ETDRS charts at 4 m. At the same time, monocular and binocular VA at different contrasts (100%, 50%, and 10%) was evaluated. The VA evaluations at different contrasts were performed with the FrACT3.9.9a version of the Freiburg Acuity Test software package, which has been employed in several previous studies [17–19]. In addition, binocular defocus curves with distance correction were measured at different vergences from +1 to −3.0 D in 0.5 D steps. Photopic conditions for all measurements were 85 cd/m.

Finally, patients also completed a five‐points Likert scale questionnaire reporting about satisfaction at different distances, perceived halo, and if they would operate again with the same lens.

### 2.3. IOLs of the Study

The TECNIS Synergy IOL incorporates both diffractive EDoF and bifocal technology on the posterior optic to provide a range of vision at different distances. In this design, intermediate focus is provided from the EDOF component while the near and distance focal points are provided by the bifocal component of the lens. Different authors have described this IOL as hybrid technology IOL [9]. In addition, the biconvex optic has a wavefront‐designed aspheric anterior surface that also provides a negative SA of − 0.27 μm to compensate for the positive spherical aberration of the cornea [13, 20].

The TECNIS Synergy is made of hydrophobic acrylic material with ultraviolet filtration and a refractive index of 1.47. It is a single‐piece IOL with C‐loop haptics and a square edge [21]. The total diameter is 13.0 mm, and the optic diameter is 6.0 mm. The IOL is available from +5.0 diopters (D) to +34.0 D in 0.5 D increments.

As previously mentioned, the TECNIS Synergy Toric II is the toric version of TECNIS Synergy and is also an aspheric design with similar material, refractive index, diameters, and range of power. The lens presents astigmatism correction from 1.00 to 2.60 D at the corneal plane.

### 2.4. Statistical Analysis

The calculation of the required sample size was based on monocular CDVA as the primary outcome variable. A difference of 0.2 logMAR units was considered clinically significant, and a standard deviation of 0.05 was assumed based on previously published data [22]. The sample size was calculated using a two‐sided independent‐samples *t*‐test, with an alpha level of 0.05 and a statistical power of 0.80. Based on these assumptions, a minimum of 25 eyes per group was required.

Statistical analysis was performed using SPSS for Windows V.20.0 (SPSS Inc., Chicago, IL, USA). The Kolmogorov–Smirnov test was used to evaluate the normality of the data distribution. Variables with a normal distribution were compared using the Student′s *t*‐test for independent samples, while the Mann–Whitney *U* test was used for nonnormally distributed variables. Differences were considered statistically significant when the *p* value was < 0.05.

## 3. Results

A total of 114 eyes from 57 patients were included in the study. One group of patients underwent bilateral implantation of the TECNIS Synergy IOL, whereas the other received the toric version, the TECNIS Synergy Toric II. Preoperative characteristics of both groups are shown in Table 1. Three months after surgery monocular UDVA was 0.02 ± 0.07 logMAR for the nontoric group and 0.08 ± 0.11 logMAR for the toric group (*p* = 0.02). Binocular UDVA were: −0.05 ± 0.06 and −0.02 ± 0.10 logMAR (*p* = 0.13) for the nontoric and the toric group, respectively. At the same time, no intra or postoperative complications were found.

**TABLE 1:** Preoperative characteristics of the eyes included in the study.

	Number of eyes (patients)	Sex (M/F)	Mean age (years)	Photopic pupil (mm)	Axial length (mm)	IOL power (D)	Sphere (D)	Cylinder (D)	Spherical equivalent (D)	CDVA (logMAR)	UDVA (logMAR)
Synergy	52 (26)	3/23	67.44 ± 8.30	3.76 ± 0.67	23.18 ± 1.05	22.15 ± 2.29	1.6 ± 2.21	−0.39 ± 0.42	0.87 ± 2.21	0.09 ± 0.11	0.51 ± 0.39
Synergy Toric II	62 (31)	10/21	72.55 ± 7.21	3.90 ± 0.91	23.67 ± 1.02	22.27 ± 2.05	−0.05 ± 2.53	−1.20 ± 1.00	−0.65 ± 2.48	0.13 ± 0.17	0.66 ± 0.46
*p* value			0.01	0.26	0.05	0.01	0.05	< 0.001	0.01	0.13	0.09

*Note:* Values provided are mean ± standard deviation. IOL, intraocular lens.

Abbreviations: CDVA, corrected distance visual acuity; UDVA, uncorrected distance visual acuity.

Three months after the surgery, the mean sphere was 0.09 ± 0.23 D (range −0.50 to +0.50 D) and 0.23 ± 0.36 D (range −0.50 to +0.75 D), the mean cylinder was −0.07 ± 0.25 D (range −1.00 to 0.00 D) and −0.04 ± 0.13 D (range −0.50 to 0.00 D), and the spherical equivalent of the manifest refraction was 0.06 ± 0.26 D (range −0.50 to 0.50 D) and 0.21 ± 0.38 D (range −0.75 to 0.75 D) in the spherical IOL group and the toric IOL group, respectively.

**FIGURE 1:**
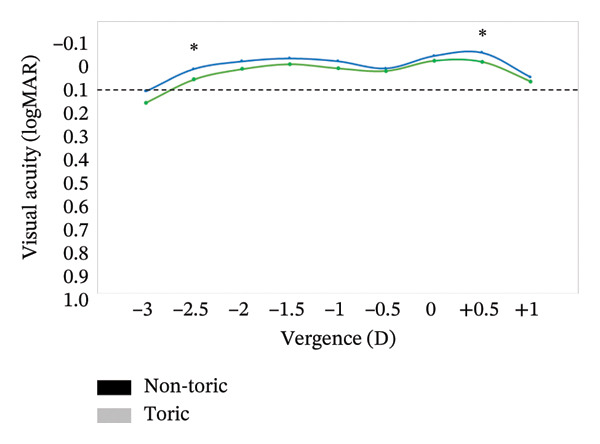
Binocular defocus curves of both groups (nontoric and toric) 3 months after bilateral cataract surgery.

**FIGURE 2:**
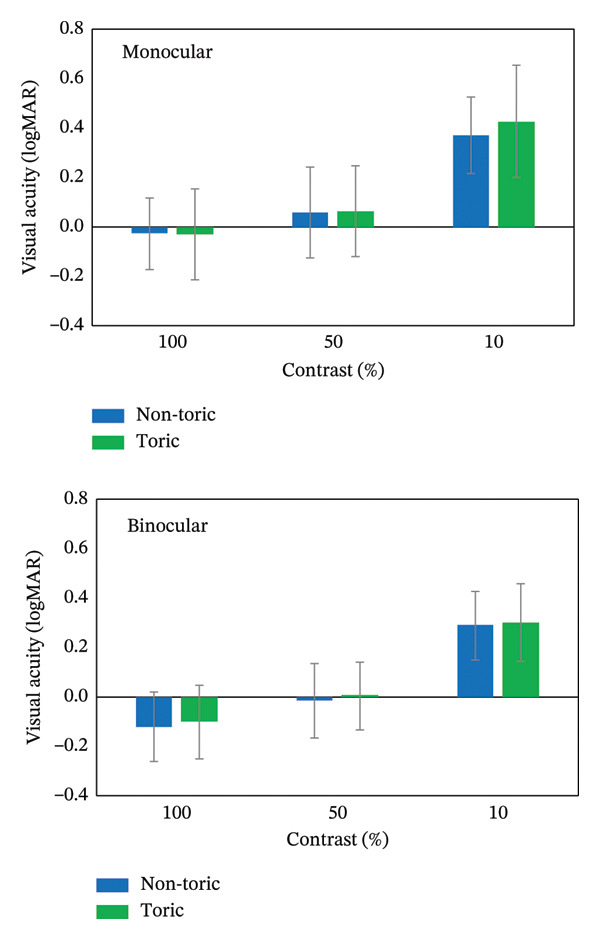
Monocular (top) and binocular (bottom) corrected visual acuity at different contrasts of both groups (nontoric and toric) 3 months after bilateral cataract surgery.

Binocular defocus curves for both groups are shown in Figure 1. Statistically significant differences were found only for +0.50 and −2.50 D of object vergence, and VA was > 0.1 logMAR (> 0.8 decimal) between +1.0 and −2.5 D of vergence for both groups. In addition, monocular and Binocular VA at different contrasts for both groups are shown in Figure 2. Both in monocular and binocular conditions, VA decreased as far as contrast decreased in both groups. However, no statistically significant differences were found between both groups for any situation analyzed (*p* > 0.05).

**FIGURE 3:**
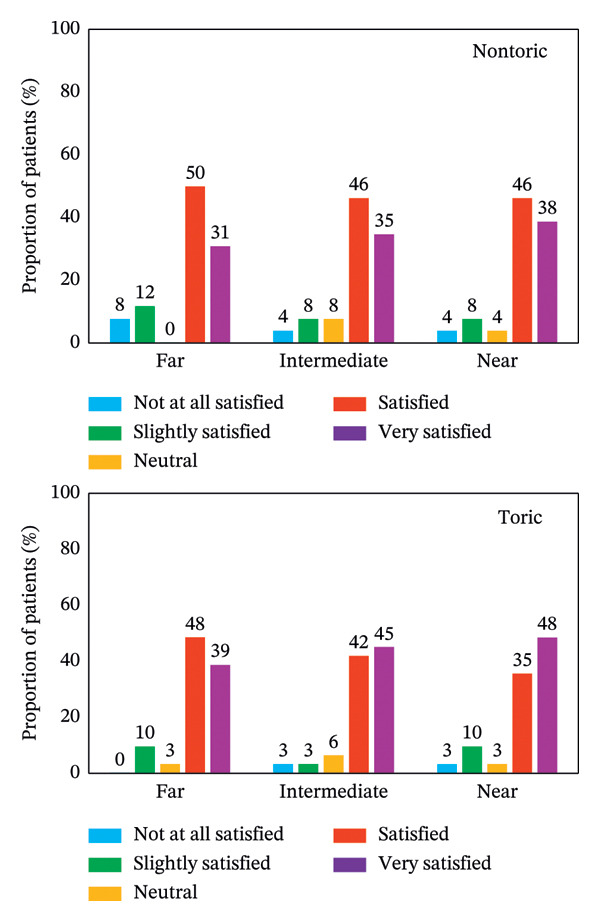
Proportion of patients in both groups (nontoric and toric) reporting satisfaction with vision at far, intermediate, and near distances.

**FIGURE 4:**
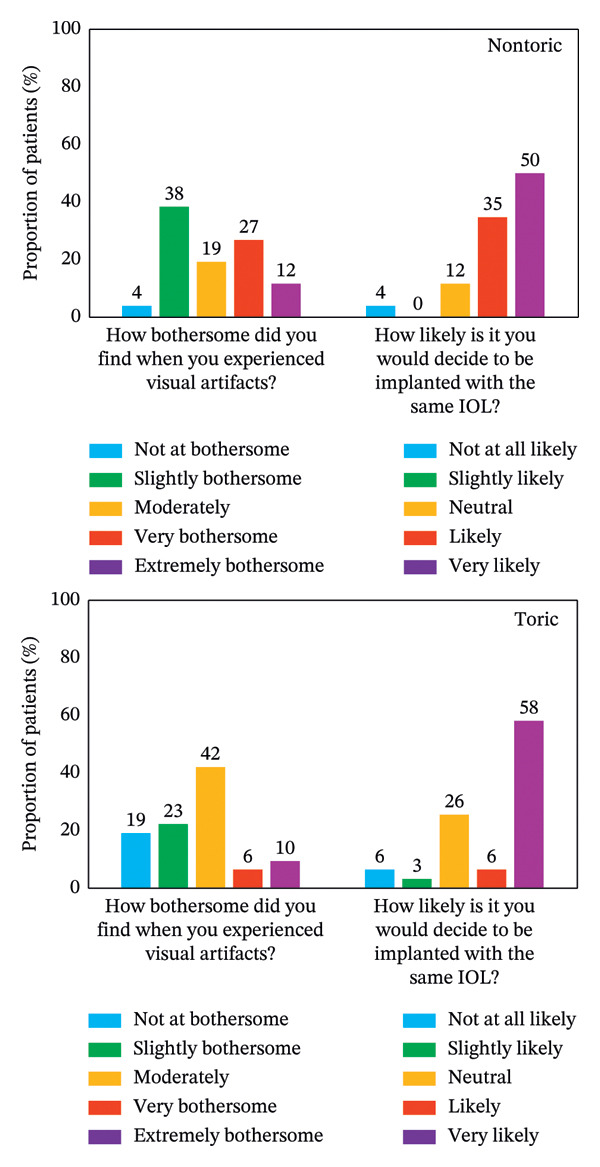
Proportion of patients in both groups (nontoric and toric) reporting the presence of visual artifacts and if they would operate again with the same lens.

For the analysis of the questionnaires, the results related to “satisfaction at different distances” are shown in Figure 3. At far, intermediate, and near distances, 81%, 81%, and 84% of patients in the nontoric group and 87%, 87%, and 83% in the toric group reported being “satisfied” or “very satisfied” three months after the surgery, respectively. Finally, the results of the questions about the “presence of halos” and if “they would operate again with the same lens” are shown in Figure 4. The 42% of patients in both groups reported that halos were “not at all bothersome” or “slightly bothersome. At the same time, 4% and 9% of patients in the nontoric and the toric group, respectively, responded that they were “not at all likely” or “slightly likely” would operate again with the same lens.

## 4. Discussion

Trifocal IOLs are designed to provide satisfactory vision at different distances and minimize patient’s spectacle dependence. The great majority of current trifocal lenses preset diffractive designs and although good results have been shown since the launch of first models, novel designs aiming to improve the visual quality are continuously available for surgeons. In the current work, we have analyzed and compared the nontoric and the toric version of an IOL that feature a hybrid presbyopia‐correcting IOL, combining multifocal and EDOF technologies within a proprietary diffractive surface.

In the current study, the lenses analyzed provide a satisfactory restoration of VA. In the current study, the nontoric and toric group showed a mean postoperative VA of around 0.0 logMAR (1.0 decimal). Our results were comparable, at least, to those of previous studies that considered the nontoric version of the Synergy IOL [8, 9].

Performing defocus curves in patients after IOL implantation shows the patient’s VA at different object distances and allows to analyze the IOL’s capability to restore VA not only at far distance. In Figure 1, it is possible to observe that both the nontoric and the toric groups achieved satisfactory VA results from far to near distances of vision. The defocus curves for both groups were similar, and statistically significant differences at +0.50 and −2.5 D (see Figure 1) do not seem to be clinically relevant. It is possible to observe that patients with both lenses reach a minimum VA of at least 0.1 logMAR (0.8 decimal) from +1.0 D to −2.50 D, restoring VA from far to near distances. In a similar previous work comparing a different trifocal design and its toric version, we found similar results, with the only difference being that the VA at the +1 vergence was better in the current work [5]. In addition, other authors showed similar results between toric and nontoric trifocal platforms [4, 23]. Hence, this could be understood as meaning that preoperative astigmatism does not worsen the potential of these trifocal lenses if they are properly implanted.

In addition to the reported outcomes, analysis of the defocus curves revealed that VA at +0.50 D of vergence was comparable to that at 0.00 D. In this study, and in accordance with the manufacturer’s recommendations, the first positive power suggested by the IOL calculation software was selected to target emmetropia. These findings suggest that the implanted lenses may tolerate a slight hyperopic shift without compromising distance vision. This shift may correspond to an underutilized region of the defocus curve; therefore, a slight adjustment in spherical power could also enhance near VA or reduce the preferred near working distance. Hence, it could be said that precise IOL power selection is essential to maximize visual outcomes, particularly for intermediate and near vision.

Considering that multifocal toric platforms are complex and must distribute the light for achieving multifocality and compensating both meridians, we analyzed VA at different contrasts for the two groups, both in monocular and binocular conditions. As it was mentioned before, in Figure 2, it is possible to observe that VA decreased when the contrast was lower but in a similar way for the nontoric and the toric groups. Although the IOL platform was different, these results show a similar trend as in a previous study of our research group in which the visual performance was analyzed in both trifocal IOL and its toric version [19]. At the same time, it could be noted that the impact of reducing the contrast on VA results is higher for monocular conditions in both groups. Nevertheless, considering the different ways for obtaining multifocality, it should be of clinical interest to analyze whether the available multifocal platforms perform under different contrast levels. This may help to project potential visual quality outcomes besides defocus curves under photopic and high‐contrast conditions.

Alongside VA outcomes, we also analyzed subjective patients’ responses after the surgery. We asked for their satisfaction at different distances (Figure 3), for the impact of halos, and if they would be operated on again with the same IOL (Figure 4). In Figure 3, it is possible to see that > 80% of patients in both groups were “satisfied” or “very satisfied” with their vision. While results were similar for near vision, for intermediate and far distances, the results showed a slight trend (81% vs 87% in both distances) to better results for astigmatic patients. Since clinical outcomes showed to be similar, this may be due to preoperative visual expectations of patients with astigmatism vs patients without astigmatism. Nevertheless, this patient’s profile should be properly analyzed to correlate satisfaction in particular groups of patients in future studies.

When analyzing the halo effect (Figure 4), we observed a range of symptom severity in both groups. A substantial proportion of patients reported some degree of bother from halos, including 12% in the nontoric group and 10% in the toric group who described them as extremely bothersome, while 42% of patients in each group reported a low impact from this photic phenomenon. Despite the methodology for analyzing the impact of halos being different and that the toric version of the Synergy platform was not considered, Han et al. also found that around 45% of patients showed discomfort related to halos after the surgery [24]. Hence, the results show that these diffractive multifocal IOLs may show some visual disturbances such as halos after the surgery for a certain group of patients, and this may be related to the scattering effect of the lens. As was suggested in a previous study, this increased light scattering effect may be related to the higher step height applied in the chromatic aberration–correcting technology of this platform [25]. Therefore, despite excellent restoration of VA at all distances, it is essential for surgeons to provide thorough counseling regarding potential dysphotopsias with these lenses. This may result in a significantly improved patient experience, as despite the reported occurrence of halos (Figure 4), only 4% with nontoric IOLs and 9% of patients with toric IOLs indicated they “not at all likely” or “slightly likely” would operate again with the same lens.

One limitation of the present study is its nonrandomized design; however, lens allocation was based on clinical indication, with toric IOLs implanted in eyes with clinically significant corneal astigmatism and nontoric IOLs in eyes without relevant astigmatism. Although this reflects routine clinical practice, it may introduce inherent differences between groups that cannot be fully controlled despite comparable baseline characteristics.

In addition, the follow‐up period was limited to 3 months, which allows assessment of early visual and refractive outcomes but not long‐term stability. Over time, changes in the lens–capsular bag complex may occur, particularly in toric IOLs, where subtle rotational shifts can affect refractive outcomes. Accordingly, further studies with longer follow‐up are warranted, with special attention to IOL platform performance and rotational stability.

In conclusion, consistent with other trifocal IOLs currently available, both IOL models exhibited a certain degree of postoperative visual disturbances. Nonetheless, the clinical performance of the lenses was excellent and comparable between the trifocal and trifocal toric designs. When the toric variant is accurately aligned, it provides effective correction of preoperative astigmatism, thereby offering visual quality and a level of spectacle independence that are on par with those achieved by the nontoric counterpart.

## Funding

This study was supported by an Investigator Initiated Study Grant by Johnson & Johnson.

## Disclosure

One of the references cited in this manuscript is linked to the PhD thesis available at: https://docta.ucm.es/rest/api/core/bitstreams/45b9a50b-2848-480b-afa4-1dc9fc39750a/content.

## Conflicts of Interest

The authors declare no conflicts of interest.

## Data Availability

The datasets generated and/or analyzed during the current study are not publicly available but are available from the corresponding author on reasonable request.
